# DIABEO System Combining a Mobile App Software With and Without Telemonitoring Versus Standard Care: A Randomized Controlled Trial in Diabetes Patients Poorly Controlled with a Basal-Bolus Insulin Regimen

**DOI:** 10.1089/dia.2020.0021

**Published:** 2020-12-07

**Authors:** Sylvia Franc, Hélène Hanaire, Pierre-Yves Benhamou, Pauline Schaepelynck, Bogdan Catargi, Anne Farret, Pierre Fontaine, Bruno Guerci, Yves Reznik, Nathalie Jeandidier, Alfred Penfornis, Sophie Borot, Lucy Chaillous, Pierre Serusclat, Yacine Kherbachi, Geneviève d'Orsay, Bruno Detournay, Pierre Simon, Guillaume Charpentier

**Affiliations:** ^1^ Department of Diabetes, Sud-Francilien Hospital, Corbeil-Essonnes, and Centre d'étude et de Recherche pour l'Intensification du Traitement du Diabète (CERITD), Evry, France.; ^2^ Department of Diabetology, Metabolic Diseases and Nutrition, CHU Toulouse, University of Toulouse, Toulouse, France.; ^3^ Department of Diabetology, Pôle DigiDune, University Hospital, Grenoble, France.; ^4^ Department of Nutrition-Endocrinology-Metabolic Disorders, Marseille University Hospital, Sainte Marguerite Hospital, Marseille, France.; ^5^ Department of Endocrinology and Diabetes, University Hospital, Bordeaux, France.; ^6^ Department of Endocrinology, Diabetes and Nutrition, University Hospital, Montpellier, France.; ^7^ Department of Diabetology, University Hospital, Lille, France.; ^8^ Endocrinology-Diabetes Care Unit, University of Lorraine, Vandoeuvre Lès Nancy, France.; ^9^ Department of Endocrinology, University of Caen Côte de Nacre Regional Hospital Center, Caen, France.; ^10^ Department of Endocrinology, Diabetes and Nutrition, CHU of Strasbourg, Strasbourg, France.; ^11^ Professor at the University Paris-Sud, University Paris-Sud, Orsay, France.; ^12^ Centre Hospitalier Universitaire Jean Minjoz, Service d'Endocrinologie-Métabolisme et Diabétologie-Nutrition, Besançon, France.; ^13^ CHU de Nantes–Hospital Laennec, Saint-Herblain, France.; ^14^ Endocrinology, Diabetology and Nutrition, Clinique Portes du Sud, Venissieux, France.; ^15^ Sanofi-Diabetes, Gentilly, France.; ^16^ Voluntis, Suresnes, France.; ^17^ CEMKA-EVAL, Bourg-la-Reine, France.; ^18^ National Association of Telemedicine, Evry, France.

**Keywords:** Glycemic control, HbA_1c_, Hypoglycemia, Insulin therapy, Telemedicine

## Abstract

***Background:*** The DIABEO^®^ system (DS) is a telemedicine solution that combines a mobile app for patients with a web portal for health care providers. DS allows real-time monitoring of basal-bolus insulin therapy as well as therapeutic decision-making, integrating both basal and bolus dose calculation. Real-life studies have shown a very low rate of use of mobile health applications by patients. Therefore, we conducted a large randomized controlled trial study to investigate the efficacy of DS in conditions close to real life (TELESAGE study).

***Methods:*** TELESAGE was a multicenter, randomized, open study with three parallel arms: arm 1 (standard care), arm 2 (DIABEO alone), and arm 3 (DIABEO+telemonitoring by trained nurses). The primary outcome assessed the reduction in HbA_1c_ levels after a 12-month follow-up.

***Results:*** Six hundred sixty-five patients were included in the study. Participants who used DIABEO once or more times a day (DIABEO users) showed a significant and meaningful reduction of HbA_1c_ versus standard care after a 12-month follow-up: mean difference −0.41% for arm 2—arm 1 (*P* = 0.001) and −0.51% for arm 3—arm 1 (*P* ≤ 0.001). DIABEO users included 25.1% of participants in arm 2 and 37.6% in arm 3. In the intention-to-treat population, HbA_1c_ changes and incidence of hypoglycemia were comparable between arms.

***Conclusions:*** A clinical and statistically significant reduction in HbA_1c_ levels was found in those patients who used DIABEO at least once a day.

## Introduction

Self-management of diabetes is critical for minimizing the risk of macrovascular and microvascular complications.^1,2^ However, self-managed glycemic control is often suboptimal, particularly for diabetic patients under intensive basal-bolus insulin regimens.^3,4^ Among others, many diabetic patients struggle in daily life to calculate and inject appropriate doses of basal and/or meal insulin, leading to episodes of hypo or hyperglycemia. Such inconvenience is aggravated by the frequent burden of their daily routine, including irregular activities and unexpected physical activity, as well as the difficulty of complying with prescheduled medical visits, without interfering with the needs of daily working activities.

The DIABEO^®^ system (DS) is a class IIb CE-marked medical device in Europe, which has been created to overcome some of the above hurdles.^5–9^ DS is a telemedicine solution that combines a mobile app for patients (available on Android or iOS operating systems) with a web portal for health care providers that allows real-time monitoring of basal-bolus insulin therapy as well as therapeutic decision making of insulin treatment in BB-treated patients.

DIABEO uses a validated algorithm to calculate insulin doses as a function of the glucose target defined by the physician, as well of the carbohydrate intake, current glycemia, and anticipated physical activity reported by the patient. The automatic algorithm ensures the adjustment of the insulin doses of bolus and basal insulin injections, or basal pump rates, when plasma postprandial or fasting glucose levels are off target.

A previous interventional pilot study (TELEDIAB-1) conducted in 17 hospitals in France investigated the efficacy of DS in 180 poorly controlled subjects with type 1 diabetes.^7^ A significant reduction in HbA_1c_ levels (−0.91%, *P* < 0.001) was observed in patients using DIABEO combined with short teleconsultations with diabetologists every 2 weeks.^7^

Real-life studies have shown a very low rate of use of mobile health apps by patients.^10^ In type 2 diabetes, a recent real-life study showed that only 42% of participants actively used the My Dose Coach™ digital tool.^11^ Therefore, we conducted a randomized controlled trial of the efficacy of DS versus standard care in conditions close to real life.

The protocol of the TELESAGE study has been published elsewhere.^12^ In this study, we present baseline data and outcome measures at 12-months of follow-up.

## Participants and Methods

### Participants

Participants were eligible for enrollment if they were adults with type 1 and type 2 diabetes, who were poorly controlled with intensive insulin therapy, delivered by multiple daily injections or by continuous subcutaneous insulin injection (two HbA_1c_ values were ≥8%; one from less than 3 months and the other of more than 1 month before inclusion). Patients had to be treated with insulin analogs according to a basal-bolus regimen for at least 1 year and were performing self-monitoring of blood glucose (at least two glucose measurements per day).

### Trial design

TELESAGE was a 12-month, multicenter (95 public and private sites), double-randomized, open-label trial with three parallel arms, which has been conducted in real-life (pragmatic) conditions in France. The study protocol^12^ was designed by the Centre d'Étude et de Recherche pour l'Intensification du Traitement du Diabète (CERITD; a nonprofit clinical translational research center located in Corbeil Hospital, Corbeil-Essonnes, France). The DS, which combines a mobile app for patients with a web portal for health care providers, was provided by Voluntis (Suresnes, France). The trial was registered on ClinicalTrials.gov (Identification No. NCT02287532).

The eligible patients were randomized 1:1:1 into three arms: arm 1 (standard care), arm 2 (DIABEO alone), and arm 3 (DIABEO+telemonitoring delegated by the diabetologists to a nursing staff). A first cluster randomization was performed at a regional level as follows: (i) six regions included patients in arms 1 and 2 and (ii) six other regions included patients in arms 1 and 3 (see Results section). A second randomization was carried out in each studied region to allocate patients in the selected groups.

The protocol for delegating telemonitoring to the nursing team (arm 3) starts with the investigator physician who sets: (i) glycemic targets and associated treatment, (ii) alarm values that trigger nursing actions, and (iii) values for patient's self-adaptations.^12^ Then, a reference nurse initiates the patient to the use of the DIABEO app on his smartphone. The patient enters relevant data (glycemia, physical activity, and ingested carbohydrates) and DIABEO calculates the insulin dose (an eventual dose adaptations). These data are sent every 2 h to a platform that is continuously visible by the reference nurse and the investigator. Automatic messages containing analytical data are produced every night. These messages are analyzed by the reference nurse during the morning of each working day. Finally, the investigator receives the data from the patients and the reports from the nurses.

Following a screening period of 10 days, the main study period lasted 12 months, with an optional extension period of at least 12 additional months. If desired, patients from the control group could start using DIABEO after 12 months (see study design in reference 12).

### Ethics approval of the study

The clinical trial was conducted in accordance with the Declaration of Helsinki and Good Clinical Practice guidelines and in accordance with the law “Informatique et Libertés” relative to the processing of personal data in the field of health (Act of 6 January 1978, amended by Law No. 2004-801 of August 6, 2004).

The study started after the sponsor had obtained the favorable opinion of the Ethics Committee (CPP, Comité de Protection des Personnes; Committee for People Protection) of La Pitié-Salpetrière Hospital (Ile de France VI) and the authorization of the French ANSM (Agence Nationale de Sécurité du Médicament; National Agency for Drug Safety). The study was registered under ANSM number: 2012-A00072-41. The sponsor communicates all serious and unexpected adverse events to the CPP and the ANSM.

### Outcome measures

Effectiveness outcomes included: (i) the mean change in HbA_1c_ from baseline to 12 months (primary endpoint), (ii) DIABEO usage rates (defined as the mean number of daily calculations of prandial insulin doses extracted from the DIABEO electronic database during the month before the last HbA_1c_ dosage), (iii) predictive factors of both, glucose control improvement and DIABEO use, and (iv) occurrence of hypoglycemia (safety outcome; details of evaluation criteria can be found elsewhere^12^).

An independent “Hypoglycemia Adjudication Committee” validated the classification of all declared hypoglycemic episodes. A severe hypoglycemic episode means that the patient required the indispensable assistance of a third person. A symptomatic hypoglycemic episode refers to those symptoms of hypoglycemia associated with rapid recovery after self-administration of sugar. Quality of life was evaluated using a slightly modified EQ-5D (EuroQol-five dimension) questionnaire.^13^

### Statistical analysis

#### Sample size

Statistical analysis predicted an initial sample size of 531 participants to achieve ≥90% power in detecting a difference with an outcome of 0.5% with an estimated standard deviation of 1.2% (assuming the rate of nonevaluable patients at about 15%, and with a two-sided alpha of 0.025). Considering a randomization by cluster (one cluster being a region), the intracluster correlation coefficient was estimated to be 0.005 and the inflation factor was 1.3. Then, a total of 696 participants was required (with an average of 58 participants per region and 232 participants per arm overall).

#### Comparability of randomized groups

The comparability of study groups was verified on the basis of distribution parameters.^14^ A primary analysis (ANCOVA covariance model, adjusted by the baseline HbA_1c_ value) was performed on the HbA_1c_ change from baseline to 1 year of follow-up. The main model was also adjusted on “utilization (Y/N)” and the interaction between this covariable and the results was tested.

#### Post hoc exploratory analyses

The determinant factors of “DS usage” were tested using logistic regression models (all covariates were tested simultaneously). Subgroup analyses were performed on those participants who used DIABEO to calculate bolus doses at least once a day (DIABEO users) or at least twice a day.

## Results

**FIG. 1.**
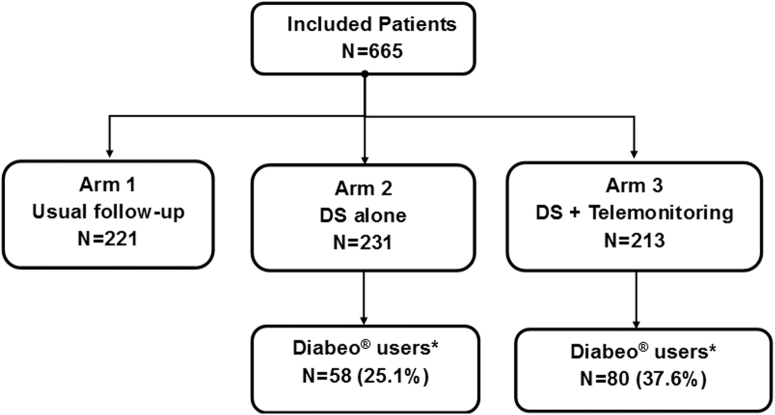
Study design and patient disposition. *DIABEO^®^ users, participants calculating prandial insulin doses with DIABEO at least one time per day during the month before the last HbA_1c_ dosage.

A total of 665 patients (ITT population) were included by 95 participating centers between April 24, 2013 and May 19, 2016 (Fig. 1). A first randomization allocated six regions (Aquitaine, Île-de-France, Lorraine, Nord-Pas-de-Calais, Rhône-Alpes, and Languedoc-Roussillon) to include patients in arms 1 and 2 and six other regions (Alsace, Franche-Comté, Basse Normandie, Midi-Pyrénées, Pays de la Loire, and Provence-Alpes-Côte d'Azur) to include patients in arms 1 and 3 (patients were evenly distributed among different French regions). A second randomization allocated 221, 231, and 213 participants to arms 1, 2, and 3, respectively (Fig. 1).

### Baseline demographic and clinical characteristics of the included patients

Baseline characteristics of the intention-to-treat (ITT) population are presented in Table 1. The mean age was 38.5 ± 13.8 years, 48.6% were male and the majority had type 1 diabetes (91.6%). The mean diabetes duration following diagnosis was 17.4 years. The mean HbA_1c_ level was high (9.1%). Insulin was administered either by a pump (53.1%) or through multiple daily injections (47.6%).

**Table 1.** Baseline Characteristics of the Included Participants by Study Arm

	Overall,* N = *665	Arm 1,* N = *221	Arm 2,* N = *231	Arm 3,* N = *213
Sociodemographic aspects				
Age, years, mean (SD)	38.5 (13.8)	38.3 (14.6)	39.1 (13.6)	38.1 (13.4)
Males, %	48.6	49.8	42.0	54.5
Public health care program, %				
State-run health care insurance	97.1	96.4	98.7	96.2
ALD30	96.7	96.8	98.7	94.4
Complementary mutual	92.2	90.0	94.4	92.0
Socioeconomic activity, %				
Farming	0.6	0.5	0	1.4
Trade, business, craftsman	4.7	4.5	4.8	4.7
Superior executive	20.0	21.3	21.6	16.9
Intermediate executive agent	8.3	4.5	9.1	11.3
Employee	32.6	34.4	32.9	30.5
Worker	5.0	5.9	3.5	5.6
Unemployed	6.3	5.4	7.4	6.1
Retired	7.2	7.2	6.1	8.5
No professional activity	15.3	16.3	14.7	15.0
Dwelling place, %				
Rural	26.8	27.1	22.9	30.5
Urban	73.2	72.9	77.1	69.5
Presentation of diabetes				
Type of diabetes, %				
Type 1 diabetes	91.6	93.6	88.7	92.5
Type 2 diabetes	8.4	6.4	11.3	7.5
Diabetes duration, mean (SD)	17.4 (10.0)	17.8 (10.0)	17.8 (10.2)	16.6 (9.9)
History of CV disease, %				
Coronary artery disease	7.4	6.4	7.4	8.6
Myocardial infarction	5.6	2.1	8.5	6.2
Heart failure	1.1	0	2.1	1.2
Cerebrovascular disease	1.5	2.1	1.1	1.2
Peripheral artery disease	8.2	9.6	5.3	9.9
History of neuropathy, %	35.7	34.0	38.3	34.6
History of nephropathy, %	36.4	38.3	42.6	27.2
History of retinopathy, %	75.1	78.7	75.5	70.4
BMI, kg/m^2^, mean (SD)	26.0 (4.8)	26.0 (5.0)	26.1 (4.7)	25.8 (4.7)
SBP, mmHg, mean (SD)	122.8 (12.7)	122.3 (12.2)	122.0 (13.1)	124.3 (12.7)
DBP, mmHg, mean (SD)	73.4 (8.8)	72.9 (8.9)	73.3 (8.5)	74.0 (9.1)
HbA_1c_ (%), mean (SD)	9.1 (1.0)	9.1 (1.0)	9.1 (1.1)	9.1 (0.9)
Severe hypoglycemia,^a^ %	9.4	7.7	10.4	9.9
Symptomatic hypoglycemia,^b^ %	72.8	72.8	68.7	77.6
Insulin treatment (% of patients)				
Multiple daily injections	47.6	49.8	42.8	50.5
Insulin pump	53.1	50.7	58.0	50.2

Arm 1: “standard care,” arm 2: DS alone, arm 3: DS+telemonitoring and teleconsultations delegated by the diabetologists to a nursing staff.

^a^ During the 6 months preceding the inclusion.

^b^ During the 2 weeks preceding the inclusion.

ALD30, state-run health care insurance covering 30 chronic diseases, including diabetes; BMI, body mass index; DBP, diastolic blood pressure; DS, DIABEO^®^ system; SBP, systolic blood pressure; SD, standard deviation.

All three study groups were comparable (not statistically different) regarding baseline values of patient characteristics (Table 1; *P* > 0.05, univariate analysis). In particular, the study groups had the same mean HbA_1c_ levels at baseline.

### HbA_1c_

**FIG. 2.**
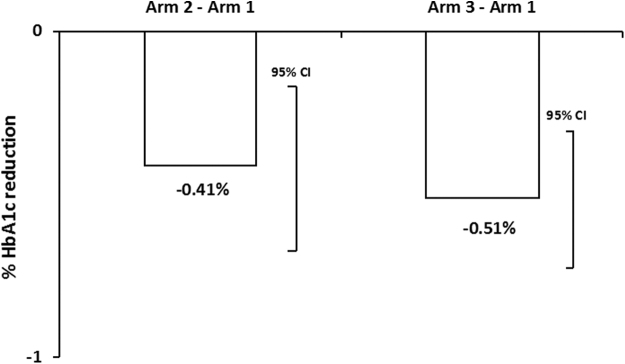
Mean changes in HbA_1c_ levels from baseline to a 12-month follow-up period were adjusted on baseline by using ANCOVA analysis. Differences in the adjusted mean changes in HbA_1c_ values of the DIABEO arms versus the control arm were highly significant (arm 2—arm 1 = −0.41% [95% CI = −0.65 to −0.16], *P* = 0.001, and arm 3—arm 1 = −0.51% [95% CI = −0.73 to −0.30], *P* ≤ 0.001). CI, confidence interval.

In the ITT population, HbA_1c_ changes (mean variations from baseline) were comparable between arms (−0.20% for arm 1, −0.34% for arm 2, and −0.26% for arm 3). A post hoc analysis in participants who used DIABEO once or more times a day (DIABEO users) showed a significant and meaningful reduction of HbA_1c_ versus standard care after 12 months of follow-up: mean difference −0.41% for arm 2—arm 1 (*P* = 0.001) and −0.51% for arm 3—arm 1 (*P* ≤ 0.001) (Fig. 2). Mean HbA_1c_ reduction values in DIABEO users from arm 3 were higher than those from arm 2, but the differences were not statistically significant (*P* = 0.448). Overall, 138 participants were DIABEO users (25.1% of participants from arm 2 and 37.6% from arm 3, Fig. 1).

In patients using DIABEO at least twice a day (13.9% of participants from arm 2 and 24.4% from arm 3) HbA_1c_ reduction was even more important (mean difference: −0.50% for arm 2—arm 1, *P* = 0.002; −0.66% for arm 3—arm 1, *P* ≤ 0.001).

### Predictive factors of glycemic control

Significant decreases in HbA_1c_ levels with respect to the control arm were observed in patients with baseline HbA_1c_ ≤9.5% (*P* < 0.029, *N* = 171 for arm 2; and *P* = 0.005, *N* = 157 for arm 3) but not in patients with baseline HbA_1c_ >9.5% (*P* = 0.826, *N* = 60 for arm 2; and *P* = 0.072, *N* = 56 for arm 3).

### Predictive factors of DS usage

An exploratory analysis showed that DIABEO usage was significantly lower in patients with baseline HbA_1c_ >9.5% (*P* = 0.036 for arm 2 and *P* = 0.005 for arm 3) or <25 years of age (*P* = 0.002 for arm 2 and *P* = 0.009 for arm 3). Interestingly, patients from arm 2 living in rural areas used DIABEO (at least twice daily) more than patients living in urban areas (23.5% vs. 10.9%, *P* = 0.030) (this was not the case for patients from arm 3). Other factors, such as gender, the type of diabetes, the socio-professional environment, and the use of pump therapy were not associated with DIABEO usage.

### DS usage during the first month of the study

**FIG. 3.**
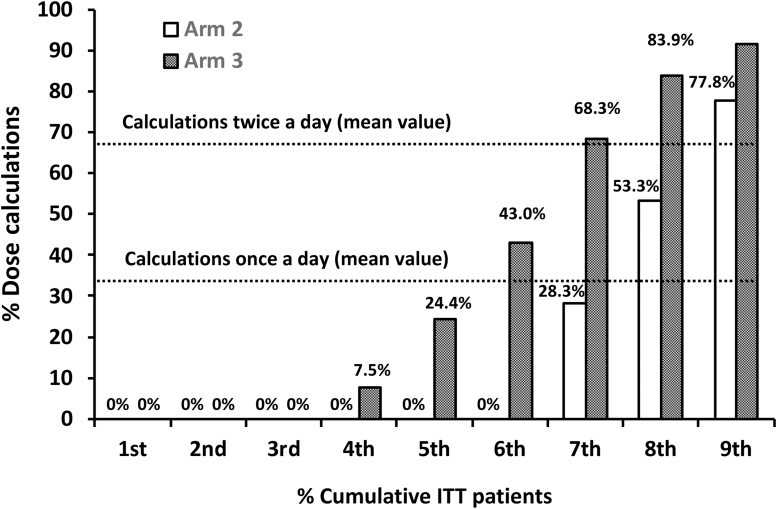
Dose calculations during the first month of the study. For each decile (Xth), % values are given as the % cumulative doses from the first decile up to the Xth decile. In arm 2, a DIABEO use of at least once a day was found for 30%–40% of patients (cumulative doses from the first decile up to the seventh to eighth decile), whereas only 20%–30% of patients used DIABEO at least twice a day (first decile up to the eighth to ninth decile). In arm 3, a DIABEO use of at least once a day was found for 50%–60% of patients (first decile up to the eighth to ninth decile), whereas only 40%–50% of patients used DIABEO at least twice a day (first decile up to the eighth to ninth decile). Zero use rates (0%) were observed in a large number of participants.

Low rates of DS use were found from the first month of the study (Fig. 3). In arm 2, a DIABEO use of at least once a day was found for 30%–40% of patients, whereas only 20%–30% of patients used DIABEO at least twice a day (Fig. 3). In arm 3, a DIABEO use of at least once a day was found for 50%–60% of patients, whereas only 40%–50% of patients used DIABEO at least twice a day.

A large number of participants never used DIABEO (Fig. 3). This was the case for 40%–50% of patients in arm 2 and for 20%–30% of patients in arm 3.

### Hypoglycemia

No significant differences between the three groups were found for the % of patients who reported at least one symptomatic hypoglycemia during the 6 months before the end of follow-up (*P* = 0.491 and 0.129 for differences between arm 2 or 3 compared with arm 1, respectively). Similarly, no significant differences among groups were found for severe hypoglycemia all throughout the study (calculated as % of patients reporting at least one hypoglycemic episode or as the incidence of hypoglycemia in patients/years).

### Quality of life

No statistically significant differences in EQ-5D scores have been found between groups (data not shown).

## Discussion

The TELESAGE trial included 665 participants eligible for the analysis, which makes it the largest (prospective and randomized) telemedicine intervention study in pragmatic condition ever conducted in diabetology. The telemonitoring service by trained nurses encouraged the use of DIABEO, and may explain its greater use in participants from group 3 with respect to those of group 2. The reduction of HbA_1c_ levels with DIABEO was strongly dependent on the frequency of its use. A statistically and clinically significant HbA_1c_ reduction was found in patients who have used DIABEO at least once a day.

At baseline, the mean HbA_1c_ level was high (9.1%) and similar to that observed in the TELEDIAB-1 study.^7^ Conversely, this mean HbA_1c_ level was higher than that observed in the French ENTRED 2007–2010 survey for the general population of French subjects with type 1 and type 2 diabetes (7.9%^15^ and 7.1%,^16^ respectively). The difference with our values can be easily explained by the fact that we have included poorly controlled patients (HbA_1c_ ≥8%), with a high incidence of diabetes complications (75.1% of the patients had retinopathy).

Patients were evenly distributed among the three arms of the study; arms that were comparable in terms of HbA_1c_ levels and other patient characteristics. DIABEO was used once a day or more by 25.1% of participants in arm 2, a proportion that increased to 37.6% in arm 3, where a team of trained nurses offered telemonitoring support to patients. Low usage rates were also found from the first month of the study. This is consistent with previous data showing that more than two-thirds of people who download a mobile health app use it only once.^10,17^

In our study, an appreciable number of participants were not familiar with the use of health apps. Due to lack of time and/or other reasons, several physicians were unable to provide technical details to the patients of arm 2. The intervention of the nursing team in arm 3 helped patients to use the application, a factor that was translated into better rates of DIABEO use.

DIABEO usage was associated with a statistically and clinically significant mean HbA_1c_ reduction *versus* standard care in arm 2 (−0.41%) and in arm 3 (−0.51%), despite the considerable attrition rates. A more important HbA_1c_ reduction was found for patients using DIABEO at least twice a day.

An exploratory analysis suggested that some factors are associated with poor use of DIABEO such as in patients with high HbA_1c_ >9.5 or young patients <25 years old, whereas others are associated with better use such people living in rural areas using DIABEO alone. These factors should be carefully considered when analyzing the effectiveness of DIABEO in real-life conditions.

On the other hand, France and several other countries are facing a serious shortage of physicians in rural areas as well as aging rural populations and impending retirement of older rural physicians.^18^ Telemonitoring solutions such as DS could represent a therapeutic improvement for those patients.

Numerous mobile applications for diabetes are available in the market, but few have clinical evaluations with results published in peer-reviewed publications, as well as regulatory clearance from the EMA or the FDA (Food and Drug Administration).^8,19,20^ The “Diabetes Interactive Diary” app software is a carbohydrate/bolus calculator, assisted by doctor/patient communication through a short message service.^21^ A randomized clinical trial showed no efficacy to improve HbA_1c_ reduction in patients with type 1 diabetes, but reduced the risk of moderate/severe hypoglycemia and improved quality of life^21^ with DS under pragmatic conditions, and TELESAGE confirmed its previously observed efficacy to improve glucose control (TELEDIAB-1 trial).^7^ Moreover, TELESAGE showed that additional benefit can be taken by coupling DIABEO to a telemonitoring service by trained nurses, an alternative to the physician-assisted telemedicine system (short teleconsultations every 2 weeks) of the TELEDIAB-1 trial.^7^

TELESAGE also provides useful suggestions for the use of telemedicine solutions in real life. Beyond measuring the conventional efficacy criteria (HbA_1c_ reduction), clinical studies should also evaluate usage rates of telemedicine solutions. Moreover, appropriate evaluations require relevant inclusion criteria. Thus, future studies are needed to identify those patients who may benefit more with telemedicine solutions. Finally, patients should be educated on the use of telemedicine solutions in clinical practice.

In the long term, DS could provide an adequate response to the lack of diabetologists in some disadvantaged geographic areas. DS could guarantee a treatment comparable to the traditional care track, or increased effectiveness when patients use the system regularly. In France, the health authorities have launched the experimental ETAPES program, which encourages and financially supports the deployment of coherent and relevant telemonitoring projects, including DS.^22^

Some other aspects of our study deserve consideration. The DS service has several strengths, including a daily data analysis. Moreover, in abnormal situations, DS sends alerts to the health care team. Delegation to the nursing staff also allows for more availability to receive patients' calls and respond to daily issues.

### Limitations of the study

There are some limitations in our study. In particular, the low usage rate of DIABEO in pragmatic conditions. Therefore, patient's support by nurses needs to be improved in future studies. The assessment of efficacy for glycemic control was performed on a population group basis, potentially ignoring site-specific factors. Finally, HbA_1c_ levels significantly decreased in patients with baseline HbA_1c_ ≤9.5% but not in patients with HbA_1c_ >9.5%. Therefore, one may expect that better DS efficacy could be obtained in a group of patients with HbA_1c_ <9.5%.

## Conclusions

A clinically and statistically significant HbA_1c_ reduction was observed in those patients who used DIABEO at least once a day. The reduction of HbA_1c_ was even more important in patients who used DIABEO at least twice a day. The inclusion of a telemonitoring service by trained nurses expanded the use of DIABEO under real-life conditions. An analysis of predictive factors suggests that DIABEO could be particularly suitable for adults and older people with mildly uncontrolled diabetes, as well as for those living in rural areas.
